# Socioeconomic position is associated with surgical treatment of open fractures of the lower limb: results from a Swedish population-based study

**DOI:** 10.1080/17453674.2020.1751418

**Published:** 2020-04-14

**Authors:** Yamin Granberg, Kalle T Lundgren, Ebba K Lindqvist

**Affiliations:** a Department of Molecular Medicine and Surgery, Karolinska Institute, Stockholm;; b Craniofacial Diseases, Karolinska University Hospital, Stockholm;; c Reconstructive Plastic Surgery, Karolinska University Hospital, Stockholm, Sweden

## Abstract

Background and purpose — High-energy trauma to the lower limbs can result in open fractures, treated by reconstructive surgery or amputation. We examined whether socioeconomic position is associated with choice of primary treatment.

Patients and methods — We performed a nationwide population-based study using the Swedish National Patient Register to identify all adult patients who between 1998 and 2013 underwent reconstruction or amputation after an open fracture below the knee. Information on socioeconomic position was collected from Statistics Sweden.

Results — Of 275 individuals undergoing surgery after an open fracture below the knee during the study period, the 1st surgery was reconstructive in 58% of the patients and amputation in 42%. The chance of having an initial reconstruction was lower for women than for men (OR 0.5, 95% CI 0.3–0.9), lower with age (OR 0.97, CI 0.96–0.99), and lower for individuals without employment compared with individuals in employment (OR 0.3, CI 0.2–0.5). Primary treatment was in women associated with family composition, whereas in men it was associated with level of education.

Interpretation — Choice of primary treatment after open fracture in the lower limb is affected by socioeconomic position including sex, age, employment, family composition, level of education, and income.

In Sweden the prevalence of open tibia fractures is around 220 per year of which around one-third are classified as Gustilo–Anderson III (Weiss et al. 2008, Tampe et al. 2014). Similar incidences of open tibia fractures have been shown in studies on other populations (Court-Brown et al. 2012).

Outcomes are poor for reconstructed and amputated patients alike, and in terms of function and pain do not necessarily differ between reconstruction and amputation (Bosse et al. 2002, Busse et al. 2007, Akula et al. 2011, Soni et al. 2012). Nearly half of patients treated for an open lower limb fracture will end up with a decreased range of motion, and little more than half of the patients are able to return to work (Busse et al. 2007, Soni et al. 2012, Barla et al. 2017). Reconstruction of the limb is easier for patients to accept, and may be preferred (Akula et al. 2011).

Scoring systems such as the Ganga Hospital Open Injury Score (GHOISS) and the Mangled Extremity Severity Score (MESS) are available to guide the treating surgeon in the decision-making process, and account for the degree of tissue damage as well as other patient-related factors (Helfet et al. 1990, Rajasekaran et al. 2015). However, the utility of such scoring systems has been questioned (Ly et al. 2008, Loja et al. 2017). Long-term outcomes also appear to be affected by patient-related factors such as socioeconomic position and personal resources (MacKenzie et al. 2006, Driesman et al. 2017).

Socioeconomic position, such as sex, level of education, income, family composition, and immigrant status, has in other healthcare areas been connected to incidence and outcome of disease (Woodward et al. 2015, Abdoli et al. 2017, Zommorodi et al. 2019). Furthermore, socioeconomic position, as determined by income and education, has been shown to affect the likelihood of undergoing operative treatment after a cruciate ligament injury in Sweden (Nordenvall et al. 2017). We examined whether determinants of socioeconomic position are associated with choice of primary treatment in patients with open fractures of the lower extremity.

## Patients and methods

We performed a population-based, nationwide register study using the Swedish National Patient Register (NPR) as well as register data from Statistics Sweden. All patients from the age of 16 and above who underwent amputation and/or reconstructive surgery for open fractures as a primary intervention in the lower extremity between January 1, 1998 and December 31, 2013 were identified through the NPR. From the same data source, information on type of injury and comorbidity by ICD-10 code, date and type of surgical procedure, hospital, county of habitation, age, and sex were collected (Table 1, see Supplementary data for a list of ICD codes). The identified individuals, being primarily treated with reconstructive surgery and/or amputation, were assumed to have extensive soft tissue damage and/or articular communication and/or contamination, therefore corresponding to Gustilo-Anderson grade III injuries.

**Table 3.** Distribution of socioeconomic factors by primary treatment. Values are frequency (%)

	Amputation	Reconstruction	All
Factor	n = 115 (42)	n = 160 (58)	n = 275
Disposable income **^a^**			
F1 (–11,527–7,963)	19 (17)	31 (19)	50
F2 (8,036–10,700)	28 (24)	21 (13)	49
F3 (10,718–14,082)	26 (23)	24 (15)	50
F4 (14,182–19,327)	15 (13)	34 (21)	49
F5 (19,355–56,991)	15 (13)	34 (21)	49
Missing	12 (10)	16 (10)	28 (10)
Employment status			
Employed	40 (35)	97 (61)	137 (50)
Not employed	63 (55)	47 (29)	110 (40)
Missing	12 (10)	16 (10)	28 (10)
Family composition			
Married/cohabiting	28 (24)	48 (30)	76 (28)
Single with child/ren	4 (3,5)	12 (7.5)	16 (5.8)
Single without child/ren	64 (56)	74 (46)	138 (50)
Living with parents	7 (6.1)	10 (6.3)	17 (6.2)
Missing	12 (10)	16 (10)	28 (10)
Level of education			
Low (≤ 9 years)	48 (42)	42 (26)	90 (33)
Middle (10–12 years)	45 (39)	65 (41)	110 (40)
High (> 12 years)	8 (7.0)	36 (23)	44 (16)
Missing	14 (12)	17 (11)	31 (11)

**^a^**Yearly, in €(1 €= 11 Swedish Krona).

The dataset was cross-referenced by the register holder, the National Board of Health and Welfare (Socialstyrelsen), with the Statistics and Longitudinal Integration Database for Health Insurance and Labour Market Studies (Swedish acronym: LISA) from Statistics Sweden. From the LISA database information was retrieved on socioeconomic variables including disposable income (the sum of income, capital gain/loss, and tax), employment, level of education, and family composition, for all study subjects. The LISA database has a high level of completeness (Ludvigsson et al. 2019). Information was collected from the year prior to the surgical treatment for the lower extremity injury.

### Statistics

Categorical variables were described with frequencies and percentages, and continuous variables with means and standard deviations (SD). Age and disposable income were analyzed both as continuous variables and as categorical variables. The quintiles of the distribution of disposable income were used to divide disposable income into 5 equal parts, with the lowest 5th referred to as F1 and the highest as F5. From records of number of years in school, the levels of education were defined as low (< 10 years), middle (10–12 years), or high (> 12 years). County of habitation was created as a binary variable, divided into urban counties, with a university hospital and level 1 trauma center, and rural counties. In the analysis, F5 was used as a reference for disposable income and the highest educational level was used as reference for level of education. When investigating association between employment and choice of treatment we excluded patients over the age 65, the general age for retirement in Sweden, as well as patients younger than 19.

Comparisons of binary or categorical variables were performed using the chi-square test or analysis of variance (ANOVA). Continuous variables were analyzed using Students t-test. A p-value smaller than 0.05 was considered statistically significant. Effect sizes were estimated using logistic regression and presented as odds ratios (OR) with 95% confidence intervals (CI). Statistical analyses were performed using Stata/IC 13 (StataCorp LP, College Station, TX, USA).

### Ethics, funding, and potential conflicts of interests

The study was approved by the Ethical Review Board of Stockholm, 2015/1174-32. The authors received no funding for the study and have no conflicts of interest.

## Results

275 individuals undergoing surgery after an open fracture in the lower limb during the study period were captured through NPR and included in the study (Table 2). The 1st surgery was reconstructive in 160 patients (58%) and amputation was performed in 115 (42%) patients. 75% were males and mean age at injury was 51 years (16–96). The most common injury was to the tibial shaft or the lower tibia. Details of socioeconomic factors are displayed in Table 3.

**Table 2.** Characteristics of the study population. Values are frequency (%)

	Amputation	Reconstruction	All
Factor	n = 115 (42)	n = 160 (58)	n = 275 (100)
Sex			
Male	77 (67)	128 (80)	205 (75)
Female	38 (33)	32 (20)	70 (25)
Age category			
16–19	3 (2.6)	11 (6.9)	14 (5.1)
20–39	18 (16)	43 (27)	61 (22)
40–59	25 (22)	53 (33)	78 (28)
60–79	28 (24)	36 (23)	64 (23)
≥ 80	20 (17)	5 (3.0)	25 (9.1)
Missing	21 (18)	12 (7.5)	33 (12)
Site of injury			
Upper tibia	13 (11)	17 (11)	30 (11)
Shaft of tibia	49 (43)	78 (49)	127 (46)
Lower tibia	21 (18)	35 (22)	56 (20)
Multiple fractures	6 (5.2)	8 (5.0)	14 (5.1)
Lower limb misc.	26 (23)	22 (14)	48 (18)

In the entire cohort, the chance of having an initial reconstruction was lower for women than for men (OR 0.5, CI 0.3–0.9), lower with higher age (OR 0.97, CI 0.96–0.99), lower for individuals without employment compared with individuals in employment (OR 0.3, CI 0.2–0.5), and lower for individuals with a low (OR 0.2, CI 0.1–0.5) or middle (OR 0.3, CI 0.1–0.8) level of education (Table 3). We found no statistically significant difference in choice of primary treatment related to lower disposable income (OR 1, CI 1–1) or to county of habitation (OR 1, CI 1–2).

For men, the chance of having an initial reconstruction was lower with higher age (OR 0.99, CI 0.97–1.0), lower for individuals without employment compared with individuals in employment (OR 0.4, CI 0.2–0.8), and lower for individuals with a low (OR 0.1, CI 0.1–0.3) or middle (OR 0.1, CI 0.1–0.4) level of education (Table 4).

**Table 4.** Associations of socioeconomic factors and reconstruction as primary treatment

	Men	Women	All
Factor	OR (95% CI)	OR (95% CI)	OR (95% CI)
Sex			
Male	N/A	N/A	Ref
Female	N/A	N/A	0.5 (0.3–0.9)
Age			
16–19	Ref	Ref	Ref
20–39	0.8 (0.2–3.5)	48 (3.6–632)	0.7 (0.2–2.6)
40–59	0.8 (0.2–3.2)	18.7 (2.5–138)	0.6 (0.1–2.3)
60–79	0.6 (0.1–2.4)	8 (1.5–43)	0.4 (0.1–1.4)
≥ 80 years	0.3 (0.04–2.1)	–	0.1 (0.01–0.3)
Disposable income ^a^			
F1	0.7 (0.3–1.7)	1.1 (0.2–8.0)	0.7 (0.3–1.7)
F2	0.5 (0.18–1.3)	0.3 (0.04–1.5)	0.3 (0.1–0.8)
F3	0.4 (0.2–1.0)	0.6 (0.1–3.5)	0.4 (0.2–0.9)
F4	1.1 (0.4–3.0)	0.9 (0.1–6.1)	1 (0.4–2.4)
F5	Ref	Ref	Ref
Employment status			
Employed	Ref	Ref	Ref
Not employed	0.4 (0.2–0.8)	0.1 (0.04–0.5)	0.3 (0.2–0.5)
Family composition			
Married/cohabiting	1.2 (0.56–2.1)	2.9 (0.8–10)	1.5 (0.84–2.6)
Single with child/ren	0.8 (0.2–3.6)	20 (2.3–183)	2.6 (0.8–8.4)
Single without child/ren	Ref	Ref	Ref
Living with parents	0.7 (0.3–2.1)	–	1.2 (0.4–3.4)
Level of education			
Low (≤ 9 years)	0.04 (0.005–0.3)	0.4 (0.1–1.7)	0.2 (0.1–0.5)
Middle (10–12 years)	0.05 (0.007–0.4)	1.5 (0.4–5.5)	0.3 (0.1–0.8)
High (> 12 years)	Ref	Ref	Ref
County of habitation			
Rural county	0.9 (0.5–1.6)	3.6 (1.2–11)	1.4 (0.8–2.2)
Urban county	Ref	Ref	Ref

**^a^** See Table 2.

For women, the chance of having an initial reconstruction was higher in 3 of the age groups (age 20–-79) compared with the youngest age group. The chance of an initial reconstruction was in women lower for individuals without employment compared to individuals in employment (OR 0.1, CI 0.1–0.5), higher for single women living with children (OR 20, 2–183) compared with single women without children, and higher in rural counties (OR 4, CI 1–11). In women there was no statistically significant association between level of education and primary treatment.

After excluding individuals under 19 years of age (13 individuals) and over 65 years of age (60 individuals), the association between employment and chance of initial reconstruction persisted (p = 0.006).

## Discussion

In this nationwide, population-based study, we found that choice of primary treatment of open fracture in the lower limb is associated with socioeconomic position. We found a sex bias favoring men in initial reconstruction rates. Furthermore, we found that the chance of initial reconstruction was associated with family composition for women, but with level of education for men.

Long-term outcomes after an open fracture of the lower limb with extensive soft tissue damage appear to be affected not only by the type of injury and treatment factors, but also by patient-related factors including socioeconomic position (MacKenzie et al. 2006). Our results suggest that socioeconomic position may also influence choice of primary treatment, thus potentially contributing 2-fold to long-term outcomes.

Socioeconomic position has in other healthcare areas been connected to incidence of disease as well as to outcomes; however, whether socioeconomic position affects choice of treatment in open lower limb fractures has not previously been investigated. This is to our knowledge the 1st study to demonstrate an association between treatment and patient factors such as sex, age, level of education, income, and family composition. As Swedish residents are entitled to the same quality of health care, medically unmotivated differences in treatment strategy should be explored further. It is important to examine the precise mechanisms by which the socioeconomic position influences the decision to undertake primary reconstruction or to amputate.

Choice of primary treatment of open lower limb fractures is a complex decision, and many factors are taken into account, not least patient compliance with postoperative restrictions and recommendations. Patient compliance in treatment, for example in secondary prevention of cardiovascular disease, has been shown to be lower in those with a low socioeconomic position (Wallach-Kildemoes et al. 2013). The reasons for this are unknown, but it is plausible that factors such as alcohol or substance abuse, closely associated with socioeconomic position, contribute to clinical treatment decisions as well as to patient involvement in those decisions. Since alcohol or substance abuse was very rarely recorded in the register used for comorbidities, we could not control for these factors.

We demonstrate a sex bias favoring men in initial reconstruction rates. Furthermore, we show that the chance of initial reconstruction is associated with family composition for women, and with level of education for men. These findings could possibly be explained by the residual confounding factors mentioned above. That patients with a higher level of education are more likely to get offered initial reconstructive surgery is not perhaps surprising; however, it is a notable finding that this association is seen only in men and not in women in our cohort. This could be due to a treatment selection sex bias, but we cannot fully explain this discrepancy from the data at hand.

With the use of the registries available in Sweden it is possible to get a high inclusion rate and a large amount of cases, despite the injuries being rare. A potential challenge when working with register data that covers a long time span is that the definition of the parameters might change over the years and not be comparable. Therefore, we have tried to limit the parameters in the study to those that have kept the same or similar definition during the full study period.

As expected, there was a higher proportion of reconstructed patients in the younger age groups. It is likely that comorbidity increases with age, and that this affects treatment decisions. We collected data for all ICD codes registered during the hospital stay, but adjustment for comorbidities such as associated trauma, cardiovascular disease, or substance abuse was impossible due to very few such registrations. However, at least 1 prior study including severely injured limbs has shown that neither severity of injury nor associated injuries affect functional outcome (Bosse et al. 2002).

Complex lower limb injuries should be referred to level 1 trauma centers managed in multidisciplinary collaboration (Naique et al. 2006, Sommar et al. 2015). In Sweden, most local or regional hospitals have some collaboration with a university hospital with access to both plastic and orthopedic surgeons. This is probably reflected by our finding that, in the entire cohort, there was no difference in the choice of primary treatment by county of habitation. That women in rural counties were more likely to be reconstructed than women in urban counties is more difficult to explain. No such association was found for men, and it is possible that family composition is a confounder in this relationship in the female subgroup.

The strengths in our study lie in the population-based study design, and in the register-based methodology, excluding the possibility of recall bias or physician selection bias. Limitations include some missing data in a few of the variables, and the retrospective study design. Missing data in the age variable are likely due to patient age not always being noted in the electronic medical records on emergency admissions of unidentified trauma patients, whereas missing data in for example disposable income is likely age-related. We did not have access to individual medical records and, as previously mentioned, residual confounding could explain some of the associations detected. We were not able to ascertain unilateral or bilateral injuries, or even the individual grades of injury. However, since all individuals received treatment with either amputation or reconstructive surgery, we deem it reasonable to assume that a majority of them had injuries corresponding to Gustilo-Anderson grade III.

Orthopedic and reconstructive plastic surgeons should be alert to the risk of undue influence in treatment selection from patient socioeconomic factors, and treatment guidelines are probably helpful tools to guide clinicians towards non-biased management.

## Supplementary Material

Supplemental Material

## Supplementary data

Table 1 is available as supplementary data in the online version of this article, http://dx.doi.org/10.1080/17453674.2020.1751418
