# Supplementary material for: Socioeconomic position is associated with surgical treatment of open fractures of the lower limb: results from a Swedish population-based study
Source: Acta Orthop. 2020 Apr 14;91(4):439–43. doi: 10.1080/17453674.2020.1751418 (PMC8023872; doi:10.1080/17453674.2020.1751418)
Supplement: Supplemental Material [file IORT_A_1751418_SM4266.pdf]

Supplementary data

Table 1. List of ICD codes

**ICD codes used for identifying reconstructive interventions**
ZZQ00 free microvascular skin transplant<br>ZZQ10 free microvascular skin and muscle transplant<br>ZZQ20 free microvascular skin muscle and bone transplant<br>ZZQ30 free microvascular muscle transplant<br>ZZQ40 free microvascular bone transplant<br>ZZR00 cutaneous flap<br>ZZR05 fasciocutaneous flap<br>ZZR10 musculocutaneous flap<br>ZZR20 cutaneous muscle and bone flap<br>ZZR30 muscle flap<br>ZZR40 muscle and bone flap<br>ZZS00 rotation plasty<br>ZZS10 transposition plasty<br>ZZS20 distant flap<br>ZZS40 V-Y or Y-V plasty<br>ZZS45 Z-plasty<br>ZZS50 tissue expansion, expander implantation<br>ZZS70 tissue expansion, expander extraction
**ICD codes used for identifying amputations**
NFG19 interposition arthroplasty hip<br>NFG99 other excision, reconstruction or arthrodesis of the hip<br>NFQ09 exarticulation hip<br>NFQ19 transfemoral amputation<br>NFQ99 other amputation or related operation on femur<br>NGQ09 exarticulation knee<br>NGQ19 transtibial amputation<br>NGQ99 other amputation or related operation on knee or tibia
**ICD codes used for trauma diagnosis groups**
S82.11 open fracture upper tibia<br>S82.21 open fracture shaft of tibia<br>S82.21 open fracture shaft of tibia<br>S82.71 multiple open fractures tibia
**ICD codes used for identifying comorbidity diagnosis**
E105, E106D, E115, E117 diabetes<br>I702, I702A, I702C atherosclerosis<br>J448, J449 chronic obstructive pulmonary disorder<br>I500, I509, I110, I130 heart failure<br>Z72, Z720, Z720A smoking<br>F10, F19 alcohol dependence<br>F01, F03, F00, G30 dementia
